# A rare intersection of Mood and Genetics: Fragile X Tremor Ataxia Syndrome (FXTAS) with Psychotic Depression

**DOI:** 10.1192/j.eurpsy.2026.11563

**Published:** 2026-07-31

**Authors:** S. M. Toparlak, M. Hussain, P. Mitter

**Affiliations:** Oxford Health NHS Foundation Trust, oxford, United Kingdom

## Abstract

**Introduction:**

FXTAS is a neurodegenerative disorder seen in older premutation (55-200 CGG repeats) carriers of *FMR1.* Females may be less affected by FXTAS due to the presence of a second X chromosome that lacks the premutation. The prevalence of FXTAS is approximately 40% in male premutation carriers, while it remains significantly lower and rare in females, affecting only about 16% of female carriers. (Cabal-Herrera et al.,2020)

Psychiatric comorbidities in FXTAS were studied by researchers and found to be common. Chi et all. mentioned that even at early FXTAS stages, patients have significant cognitive and other psychiatric symptoms, with notable gender-specific differences.

**Objectives:**

To describe a rare case of Fragile X-associated Tremor/Ataxia Syndrome (FXTAS) presenting with psychotic depression, highlighting the intersection of genetic, neurological, and psychiatric domains.

**Methods:**

The patient underwent comprehensive neuropsychiatric evaluation, including cognitive testing, MRI brain, and Fragile X premutation genetic analysis. Psychiatric symptoms were assessed using standard diagnostic criteria and managed with pharmacological and psychosocial interventions

**Results:**

MRI (Magnetic Resonance Imaging) scan in 2016 showed global involutional change in advance of the patient’s age, which was requested due to difficulties with balance, falls, visual disturbance, history of severe depression and apathy. CT head scan in 2016 showed extra-axial spaces and ventricles being generally slightly prominent consistent with atrophy. In the same year, the 111 gene panel for retinal dystrophies revealed negative results. DAT (Dopamine Transporter Scan), SPECT (Single-photon emission computed tomography) /CT scan and nerve conduction studies in 2017 both reporting as normal with no evidence of dopaminergic synaptic loss. In July 2017, after comprehensive clinical assessment by the neurology team she was diagnosed with MSA type C (cerebellar form). However, a genetic testing in May 2023 confirmed FMR1 premutation, which, taken alongside her clinical syndrome of slowly progressive late onset tremor and ataxia. This resulted in her previous diagnosis of MSA type C to be revised into a diagnosis of Fragile X Tremor Ataxia Syndrome (FXTAS).

**Image 1:**
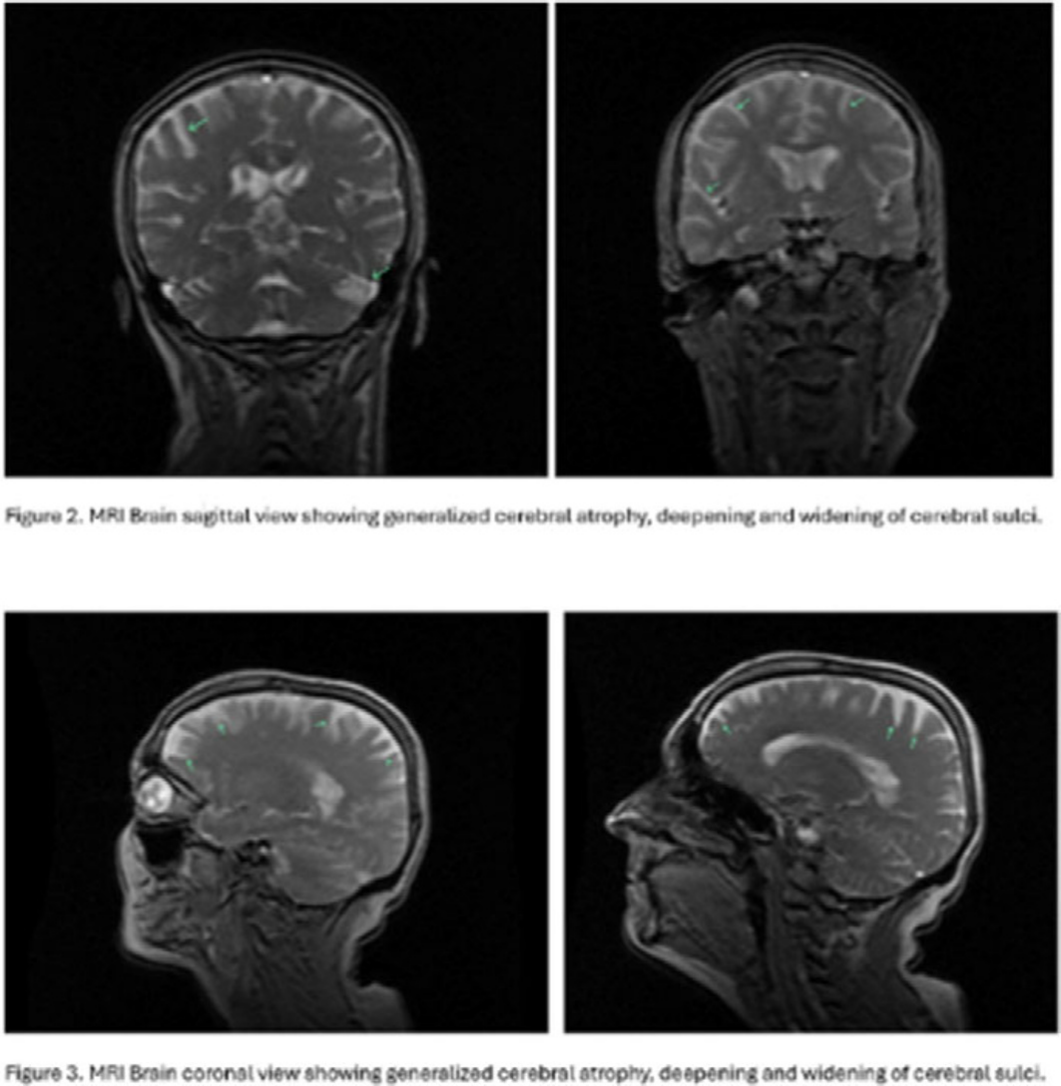
Image 1: Long description.

**Image 2:**
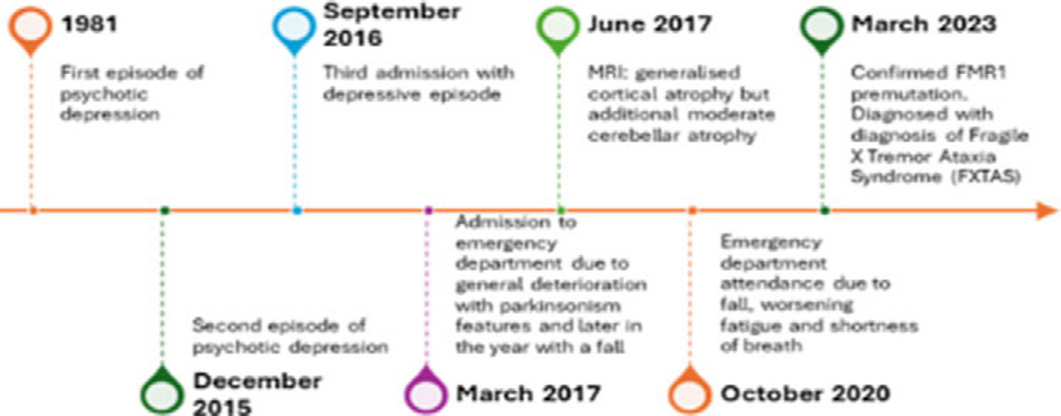
Image 2: Long description.

**Conclusions:**

FXTAS can present with late-onset psychotic depression and unusual behaviour, particularly in female premutation carriers, despite its rarity.

Neurogenetic testing could be considered in older adults with atypical psychiatric presentations, unexplained tremor, or progressive ataxia—especially with a family history of neurodevelopmental or neurodegenerative conditions. Additionally, MRI findings and neuropsychological assessment can support diagnosis by revealing cerebellar atrophy and executive dysfunction, both features of FXTAS.

**Disclosure of Interest:**

None Declared

## Long descriptions

### Image 1: Long description

A four-panel M R I brain scan display. The top two panels show coronal views of the brain. In the top-left panel, green arrows point to the upper-left cortical region and the lower-right temporal area. In the top-right panel, two green arrows point to the bilateral superior frontal sulci. The bottom two panels show sagittal views. In the bottom-left panel, three green arrows point to the frontal and parietal sulci. In the bottom-right panel, four green arrows highlight the prominent spacing between the brain folds along the superior surface. Throughout all panels, the dark spaces between the gray matter folds are noticeably enlarged, representing the deepening and widening of cerebral sulci characteristic of atrophy.

Navigate back to Image 1:.

### Image 2: Long description

The timeline consists of a central orange horizontal arrow with seven chronological markers.

* 1981 (Above axis, orange pin): First episode of psychotic depression.

* December 2015 (Below axis, green pin): Second episode of psychotic depression.

* September 2016 (Above axis, blue pin): Third admission with depressive episode.

* March 2017 (Below axis, purple pin): Admission to emergency department due to general deterioration with parkinsonism features and later in the year with a fall.

* June 2017 (Above axis, light green pin): M R I: generalised cortical atrophy but additional moderate cerebellar atrophy.

* October 2020 (Below axis, light orange pin): Emergency department attendance due to fall, worsening fatigue and shortness of breath.

* March 2023 (Above axis, dark green pin): Confirmed F M R 1 premutation. Diagnosed with diagnosis of Fragile X Tremor Ataxia Syndrome (F X T A S).

Navigate back to Image 2:.

